# Complementary Paths to Chagas Disease Elimination: The Impact of Combining Vector Control With Etiological Treatment

**DOI:** 10.1093/cid/ciy006

**Published:** 2018-06-01

**Authors:** Zulma M Cucunubá, Pierre Nouvellet, Jennifer K Peterson, Sarah M Bartsch, Bruce Y Lee, Andrew P Dobson, Maria-Gloria Basáñez

**Affiliations:** 1 London Centre for Neglected Tropical Disease Research, United Kingdom; 2 Medical Research Council Centre for Outbreak Analysis and Modelling, Department of Infectious Disease Epidemiology, School of Public Health, Imperial College London, United Kingdom; 3 Zoonotic Disease Research Center, Arequipa, Peru; 4 Department of Biostatistics, Epidemiology and Bioinformatics, Perelman School of Medicine, University of Pennsylvania, Philadelphia; 5 Public Health Computational and Operations Research, John Hopkins Bloomberg School of Public Health, Baltimore, Maryland; 6 Department of Ecology and Evolutionary Biology, Princeton University, New Jersey

**Keywords:** Chagas disease, mathematical model, vector control, etiological treatment, prevalence

## Abstract

**Background:**

The World Health Organization’s 2020 goals for Chagas disease are (1) interrupting vector-borne intradomiciliary transmission and (2) having all infected people under care in endemic countries. Insecticide spraying has proved efficacious for reaching the first goal, but active transmission remains in several regions. For the second, treatment has mostly been restricted to recently infected patients, who comprise only a small proportion of all infected individuals.

**Methods:**

We extended our previous dynamic transmission model to simulate a domestic Chagas disease transmission cycle and examined the effects of both vector control and etiological treatment on achieving the operational criterion proposed by the Pan American Health Organization for intradomiciliary, vectorial transmission interruption (ie, <2% seroprevalence in children <5 years of age).

**Results:**

Depending on endemicity, an antivectorial intervention that decreases vector density by 90% annually would achieve the transmission interruption criterion in 2–3 years (low endemicity) to >30 years (high endemicity). When this strategy is combined with annual etiological treatment in 10% of the infected human population, the seroprevalence criterion would be achieved, respectively, in 1 and 11 years.

**Conclusions:**

Combining highly effective vector control with etiological (trypanocidal) treatment in humans would substantially reduce time to transmission interruption as well as infection incidence and prevalence. However, the success of vector control may depend on prevailing vector species. It will be crucial to improve the coverage of screening programs, the performance of diagnostic tests, the proportion of people treated, and the efficacy of trypanocidal drugs. While screening and access can be incremented as part of strengthening the health systems response, improving diagnostics performance and drug efficacy will require further research.

Chagas disease (whose etiological agent is *Trypanosoma cruzi*) is endemic throughout much of Latin America and is responsible for substantial excess morbidity and mortality due to cardiac and digestive complications [1, 2]. It is estimated that the global costs of Chagas disease are US$7.19 billion per year and US$188.80 billion per lifetime [3]. In the context of Chagas disease, the 2012 London Declaration and the World Health Organization (WHO) roadmap on Neglected Tropical Diseases proposed that by the year 2020, total interruption of intradomiciliary transmission in Latin America and provision of care to all infected patients should be achieved [4]. However, recent data suggest that antivectorial programs are not being sustained in all endemic countries or regions [5]. Furthermore, treatment coverage is as low as <1% of the infected population [6], and access to care remains limited in several countries [7].

Based on clinical efficacy (measured as the ability to halt or curb clinical progression to heart complications), etiological treatment is only considered adequate for recent infections, such as acute or congenital cases, and infection in children [8]. Clinical efficacy is yet to be demonstrated in late-stage infections and in adults with advanced heart conditions [9]. (Etiological treatment is defined as trypanocidal treatment administered and monitored upon diagnosis of infection by polymerase chain reaction [PCR] and/or conventional methods.) The development of real-time PCR has made it possible to measure and monitor therapy; thus, the parasitological efficacy (sustained trypanocidal activity) of the currently available drugs has been recently tested. Two independent clinical trials have demonstrated parasitological cure in asymptomatic adult carriers with an efficacy of 88% [10] and 94% [11].

Historically, vector control has been the main strategy used to prevent Chagas disease transmission. Vector control typically relies on campaigns of indoor residual spraying (IRS) which, if done appropriately, have a long-lasting effect, even in hyperendemic settings with domiciliated vectors. Successful vector control campaigns have been carried out against the vector species *Triatoma infestans* in much of the South Cone of South America [12], and *Rhodnius prolixus* in Central America [13]. However, active intradomiciliary transmission still occurs in many endemic regions, particularly in hard-to-reach populations in isolated rural communities of Latin America [5].

Given the high (anti-)parasitological activity of trypanocidal drugs and the limited success in interrupting vector-borne *T. cruzi* transmission in several endemic regions, it is important to understand the role of other Chagas disease control strategies, both alone and in combination with vector control, to inform policy makers, researchers, and funders on future directions. Here, we examine the role of etiological treatment on Chagas disease transmission dynamics, and its potential for helping to achieve the WHO 2020 goals. Can etiological treatment reduce time to transmission interruption? To answer this question, we use a mathematical model of Chagas disease transmission based on our previous dynamic model [14] to compare time to intradomiciliary interruption of *T. cruzi* transmission in 2 scenarios: (1) deploying vector control (IRS) alone and (2) combining vector control with etiological treatment (measured as the proportion of parasitological cure (PPC) in the infected population.

## METHODS

### Transmission Dynamics Model

We expanded our deterministic model first presented in [14], which includes vector, human, and animal host populations. The model divides the vector population into susceptible (uninfected), $S_{V}$, and infectious individuals, $I_{V}$. The total vector population size is determined by a logistic equation with a carrying capacity $K_{V}$ that is related to the size of the human population considered. The human population is divided into 4 categories: susceptible (uninfected) individuals, $S_{H}$; acutely infected individuals, or those who have recently been infected with *T. cruzi*, $I_{H}^{a}$; asymptomatic individuals in the chronic indeterminate phase, $I_{H}^{i}$; and chronically infected individuals who exhibit clinical pathology ($I_{H}^{c}$). Individuals who reach parasitological cure after treatment are moved back into the susceptible population. To track age-specific seroprevalence status and clinical outcomes, the human population is age-structured, with a fixed birth rate. We assume that a variety of mammal species can act as *T. cruzi* hosts. This nonhuman host population (without explicit age-structure) is divided between$S_{R}$and $I_{R}$. The reservoir population is related to the size of the human population, as many of them are synanthropic (eg, peridomestic foraging marsupials).

We included an additional “external” force of infection (FoI) upon humans, $\lambda_{H}$, representing all other processes that take place outside the intradomiciliary cycle (eg, sylvatic transmission, oral transmission). Similarly, we included an external FoI upon susceptible reservoirs, $\lambda_{R}$. Model parameters and values (informed by the literature) are presented in Supplementary Table A. The full dynamical system and the model’s sensitivity analysis are presented in the Supplementary Methods.

### Vector Control

Vector control (IRS) was modeled by reducing vector density by a given proportion following an implementation process that was governed by 2 parameters: the final decrease in vector density and the time to reach such density. Over the implementation period, we gradually increased vector mortality and simultaneously decreased their carrying capacity. At the end of the implementation period, the vector density reached a new level under “control” conditions. While the duration of the implementation period relates to the initial effort allocated to vector control, the final reduction in vector density relates to vector control efficacy and coverage. The final modeled level of vector control ranged between 0 to 100% annual reduction in vector density. Vector control was gradually implemented to achieve its final and sustained level after 4 months ($\Delta_{VC}$) in a village with 10000 habitants.

### Etiological Treatment

The key aspect modeled for etiological treatment was the PPC, meaning the proportion of the *T. cruzi–*infected human population that effectively reached parasite clearance and subsequently moved back into the susceptible population. Notice that PPC is calculated as the product of (1) the proportion of infected individuals who are tested, p_T_; (2) the proportion of those tested with a positive test result (dependent on the diagnostic performance of the test(s) used), p_P_; (3) the proportion of those testing positive who are treated with available drugs and finish the entire treatment course, p_D_; and (4) the proportion of infected individuals who are cured (parasitological drug efficacy), p_E_, after undergoing treatment.

Treatment was modeled by setting the duration of implementation of human treatment, $\Delta_{HT}$, and the final value of PPC 1 year after treatment (ie, modeling the composite product of p_T_, p_P_, p_D_, and p_E_). During the implementation period, the PPC (modeled through the proportion of infected humans effectively treated, $P_{HT}$) is gradually increased to reach its final level, $1-\exp(-P_{HT})$, which is then maintained constant following the implementation period. We used a feasible range of PPC values based on the current characteristics of p_T_, p_P_, p_D_, and p_E_, ranging between 0 and 38% of infected humans each year. We assumed that the implementation of a treatment program in a community of this size would last for 12 months each year based on previous experiences of screening and etiological treatment programs in endemic areas [15, 16].

The details of the model with antivectorial and antiparasitic interventions are provided in the Supplementary Methods, with parameters and values listed in Supplementary Table B.

### Scenarios Under Evaluation

We evaluated the impact of vector control (IRS) alone, and in combination with etiological treatment, for epidemiological scenarios ranging from low to high endemicity (modeled by changing the vector carrying capacity, see Supplementary Table C). To facilitate ease of interpretation, we used values representing a low, medium, and high efficacy for vector control and etiological treatment impact. For vector control, the low, medium, and high efficacy values are presented as reductions in vector density by 10%, 50%, and 90%, respectively. For etiological treatment, we focus on the impact of PPC in 1% (status quo), 10% and/or 20% of the total infected population. Age structure is included so that results generate age-prevalence profiles, which are indicative of the long-term effect of control strategies against Chagas disease in human populations.

### Transmission Interruption Thresholds

The operational criteria proposed by the Pan American Health Organization (PAHO) to certify interruption of intradomiciliary vectorial transmission in endemic regions include *T. cruzi* seroprevalence <2% in children aged <5 years [17], absence of acute cases in the last 3 years, and other indicators of domestic and peridomestic triatomine infestation. All these criteria are currently under review by PAHO. Therefore, we have used the serological criterion thus far published [17] as a measure of transmission interruption.

## RESULTS

### Effect of Vector Control Alone and in Combination With Etiological Treatment on Seroprevalence in Children Aged <5 Years

**Figure 1.**
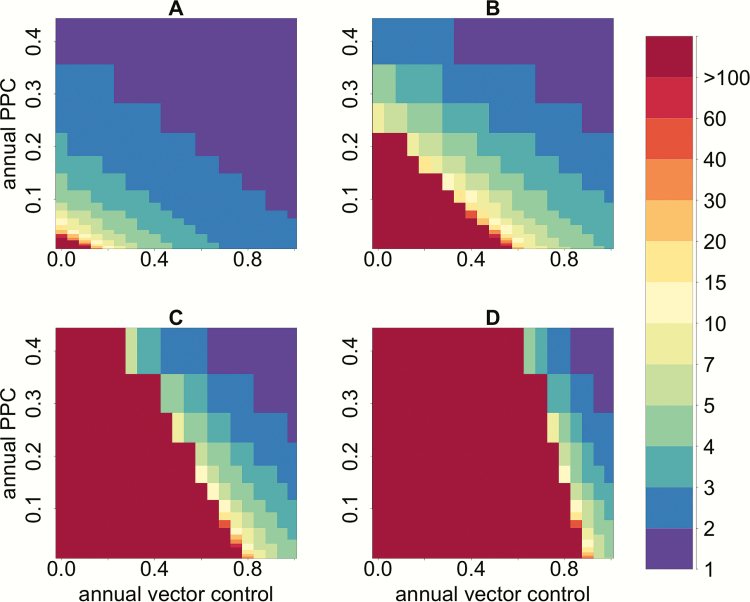
Combined impact of vector control and effective parasite clearance (measured as proportion of parasitological cure [PPC]) on years to reduce seroprevalence in children under 5 to <2%. Annual vector control defines the proportion by which vector density is reduced (0–100%); annual PPC defines the proportion of humans effectively treated, ie, the percentage of the infected human population achieving parasitological cure (0–40%). The color scale corresponds to number of years to achieve the serological criterion. The panels represent: A: low; B: moderate; C: high; D: very high endemicity levels (see Supplementary Materials).

Our model predicts that in a moderate endemicity setting, an annual 60% reduction in vector density by itself would require >45 years to reach the serological criterion for transmission interruption. By contrast, when implementing etiological treatment of infected individuals in combination with vector control, the time to achieve the seroprevalence threshold in children would be considerably reduced (Figure 1). For instance, in a moderate endemic setting, when PPC is implemented in combination with vector control, and increased to 20%, the time to reach <2% seroprevalence in children decreases from 45 to 5–6 years, a decrease by 80%–90%.

### Effect of Vector Control Alone and in Combination With Etiological Treatment on Overall Infection Prevalence in the Human, Reservoir, and Vector Populations

**Figure 2.**
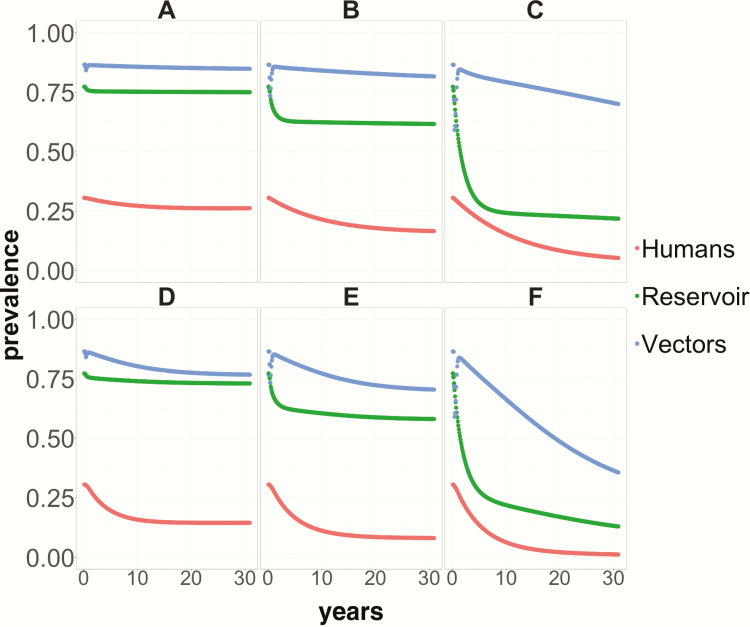
Infection prevalence in humans (red lines), nonhuman mammal hosts (green lines), and domiciliated *Trypanosoma cruzi* vectors (blue lines) over 30 years following the implementation of sustained and continuous control strategies beginning at year 1. *A*, *B*, and *C* present, respectively, prevalence trends following vector control on its own that leads to 10%, 50%, and 90% reductions in vector density. *D–F* depict prevalence trends following implementation of vector control (same reductions in vector density as above) in combination with an annual 10% proportion of parasitological cure in the population through treatment of the *T. cruzi–*infected human population.

Vector control alone must be implemented very intensively and over prolonged periods (eg, decades) to reduce markedly *T. cruzi* prevalence. In a highly endemic setting, an annual 90% reduction in vector density would take 30 years to reduce overall human infection prevalence to 5% and domiciliated triatomine infection prevalence to 70%. Contrastingly, adding etiological treatment, even at the relatively low level of 10% PPC, would lead to much faster reductions in infection prevalence, reaching, after 30 years, 7% in the human population (<60% in the reservoir population) even for moderate reductions (50%) in vector density. Higher reductions in vector density (90%), in combination with 10% PPC, would result in human and reservoir infection prevalence levels well below 1% and 15%, respectively, and <40% in the vector population (Figure 2).

### Effect of Vector Control Alone and in Combination With Etiological Treatment on Age-Specific Prevalence in the Human Population

**Figure 3.**
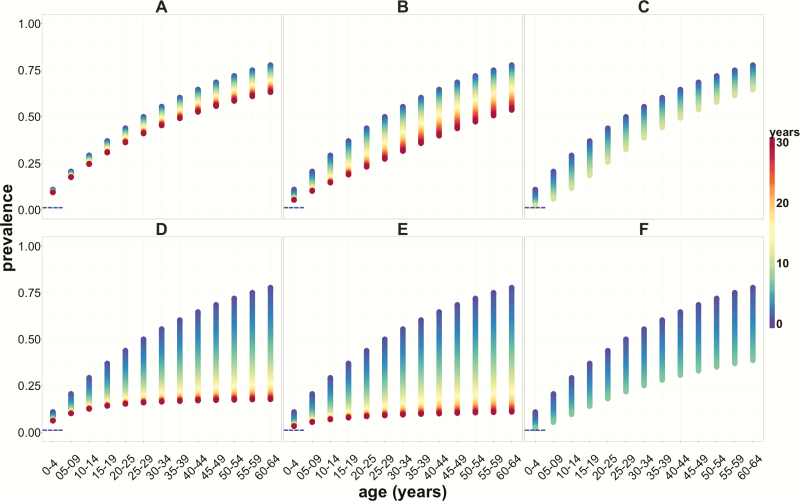
Age-specific prevalence profiles of *Trypanosoma cruzi* infection in the human population in a highly endemic setting, following over 30 years the implementation of sustained control strategies. *A*, *B*, and *C* present, respectively, age prevalence profiles corresponding to vector control and status quo treatment (1%), with annual reductions in vector density of 10%, 50%, and 90%. *D–F* depict human *T. cruzi* infection prevalence profiles following the same reductions in vector density as above in combination with etiological treatment that effects a 10% population parasite cure among the infected population annually. Horizontal blue dashed lines indicate the 2% seroprevalence threshold in children under 5. The color scale represents time, with blue representing the beginning of the intervention and red representing 30 years of sustained intervention.

The model predicts that, in a highly endemic setting, if deployed on its own, only an aggressive (90% reduction in vector density) and prolonged (>10 years) vector control strategy would reduce prevalence in children under 5 to <2%. It would take >15 years to reduce prevalence to <5% in children aged 5–15 years. If vector control strategies are implemented in combination with etiological treatment that achieves 10% PPC in the total infected human population per year, the time to achieve the former of these serological thresholds would be reduced by half, also leading to marked declines in *T. cruzi* infection prevalence in all age groups (Figure 3).

**Figure 4.**
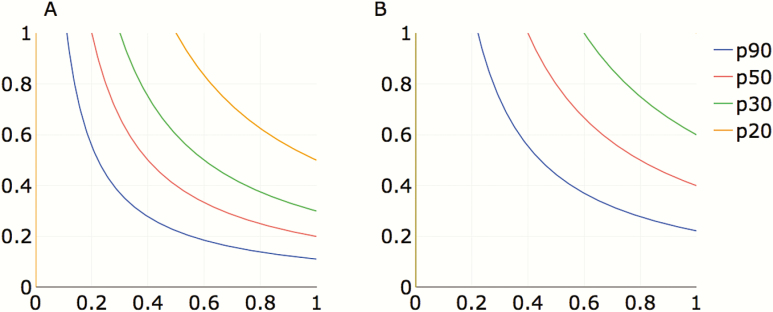
Probability of achieving 10% proportion of parasite clearance (PPC; *A*) or 20% PPC (*B*) in a *T. cruzi–*infected human population based on the combined probability of being diagnosed and treated for Chagas disase. The horizontal axis represents the combined contribution of diagnosis as a product of the proportion of infected people who are tested (pT) and the proportion of those tested with a positive test result, that is, the sensitivity of the test (pP). The vertical axis represents the combined contribution of treatment, as the product of the proportion of those testing positive who are treated with currently available drugs (pD) and respond to treatment by clearing parasites according to efficacy (pE). Colored lines represent the proportion (p) of infected people who would have to be reached by a test and treat program (90% [blue], 50% [red], 33% [green], and 20% [orange]) to achieve the desired level of effective PPC.

The role of etiological treatment on the *T. cruzi* infection dynamics in humans depends on increasing 4 potential components of effective treatment: (1) the proportion of human hosts tested for *T. cruzi* infection; (2) the proportion diagnosed as positive for *T. cruzi* infection according to the test(s); (3) the proportion of these being treated for *T. cruzi* with a trypanocidal drug; and (4) the proportion who respond to treatment by completely clearing *T. cruzi*. The net effectiveness at the population level, or PPC, is the product of all 4 components and thus effective levels can be achieved by increasing some or all of the components of the effective parasite clearance. Figure 4 illustrates some possible combinations (of diagnostic and treatment probabilities) that would lead to achieving 10% or 20% PPC, given a range of proportions of infecteds (20–90%) covered by a test and treat program.

## DISCUSSION

We have expanded a previous mathematical model of *T. cruzi* infection to explore the impact of etiological treatment combined with vector control on shortening timeframes for achieving interruption of intradomiciliary transmission of Chagas disease. Our model outputs suggest that the combination of antivectorial and antiparasitic measures would considerably decrease the incidence (measured as seroprevalence in children under 5) and overall prevalence of Chagas disease in the human population compared to antivectorial measures alone. Time to achieving the currently published serological criterion for intradomiciliary transmission interruption would be halved (when 90% of vector density is reduced and 20% PPC is sustained, Figure 1), and the benefit would extend to all hosts in the transmission system (vectors, humans and reservoirs) even when PPC is as low as 10% (Figure 2). This increase in effectiveness is achieved because treatment of infected humans reduces the number of humans infecting susceptible vectors, which, in combination with reductions in intradomiciliary vector density, would reduce the rate at which newly infected human cases arise.

It must be emphasized, however, that the modeled reductions in intradomiciliary vector density assume that IRS will be effective for triatomine vector control in all endemic areas, including the southern parts of North America (Mexico), Central America, and northern parts of South America (eg, Colombia, Ecuador, northern Peru), where transmission is still ongoing. This assumption is based on the previously documented success, in the Southern Cone of South America, in curtailing transmission by *T. infestans*. However, it is important to note that triatomine populations across the continent are heterogeneous in terms of species composition and likely also in their susceptibility to the insecticides used in IRS. The major triatomine vector of Chagas disease in Central America, Ecuador, and other areas of northern South America is *Triatoma dimidiata*. This species can be found in sylvatic, peridomestic, and domestic habitats. Nondomiciliated populations may act as sources of reinfestation and become involved in transmission to humans [18]; the vector control approaches based on IRS that have been used for *T. infestans* may not work equally well for *T. dimidiata*.

Our results highlight that treatment of patients with Chagas disease does not need to be 100% effective to reduce transmission to levels that meet the 2020 criteria; the impact of drugs with <100% efficacy can be compensated for if enough patients are diagnosed and treated. Unfortunately, there is a number of constraints in the treatment of *T. cruzi–*infected humans: (1) most asymptomatic patients do not know they are infected and do not seek treatment until they are in the chronic (determinate) phase of infection [1]; (2) drugs currently available work best in younger and more recently infected acute patients [19]; and (3) severe drug adverse events reduce the number of persons completing (adhering to) treatment [9]. This, and the prospects that we may not yet have a protective vaccine against Chagas in the near future despite efforts toward this [20, 21], highlight the need for better access to diagnosis and treatment as well as for new drugs to be developed that have fewer side effects and lead to improved compliance.

Achieving a high proportion of PPC in the population is challenging in many respects, although our results indicate that even moderate levels of etiological treatment can go a long way if combined with highly effective vector control (but see the caveats highlighted above). Based on current estimates of access to screening and etiological treatment, at <1% of infecteds [6], a diagnostic sensitivity estimated for available serological tests at 90%, a rate of abandonment of treatment at 20–30% [9], and a trypanocidal efficacy at 90% [10, 11], it would have been unrealistic to model strategies in which the effective parasite clearance in the population would have been high (thus our PPC values ranged from 0 to 38%). In fact, reaching a 40% PPC would require a proportion of screening as high as 70%. Of all the components of PPC, the major public health challenge will be increasing access to screening, currently the weakest link in the equation. Therefore, finding alternative and less costly screening strategies is paramount. Some studies have evaluated strategies of targeted screening in clusters of individuals who are at the highest risk of infection [22].

Promising progress toward the development of better diagnostics has been reported in recent years, with validation of rapid diagnostic tests (RDTs) showing similar performance to classical serological tests. RDTs have the additional benefit of requiring less trained personnel and laboratory infrastructure [23]. Table 1 summarizes potential strategies for improving the PPC.

**Table 1.** Potential Strategies for Improving the Proportion of Parasitological Cure in a Population of **Trypanosoma cruzi**–Infected Humans

Strategy	Current Situation	Potential for Improvement
Screening	<1% of populations in areas at risk of vectorial transmission are tested [6]. (In purposely designed screening programs, coverage has reached 70% [15])	Design screening campaigns and PoC diagnostics that reach the largest proportion of *Trypanosoma cruzi*–infected individuals (in addition to children); introduce screening tests in antenatal clinics and routine hospital visits in endemic areas or target populations
Diagnostic test performance	Sensitivity of serological diagnostics varies between 90% and 95% [24]	Increase sensitivity and specificity of diagnostics, especially for measuring parasitological cure
Access to and abandonment of etiological treatment	Low access to treatment among *T. cruzi–*infected individuals and high rate of abandonment because of SAEs [9, 25]	- Reduce barriers to access to treatment - Improve availability of drugs - Reduce SAEs - Increase supervision by medical professionals during treatment course
Parasitological efficacy (sustained parasite clearance)	Reported at 88% and 94% in 2 clinical trials, measured by real-time PCR [10, 11]	Develop novel drugs

Abbreviations: PCR, polymerase chain reaction; PoC, point of care; SAE, severe adverse event.

Paradoxically, although antitrypanosomal drugs can be more efficacious in children, this is the population group with the lowest prevalence, particularly in regions with long-term vector control programs in place. A further development of our model will be to evaluate different strategies for reaching the highest possible proportion of infected humans, with potential benefits for both transmission and disease burden impact. Other potential strategies to be evaluated could include increasing the age range at which diagnosis and treatment are implemented.

The current seroprevalence threshold of <2% in children aged <5 years is presently under evaluation by PAHO with the aim of extending the age class to which the criterion should be applied and/or lowering the seroprevalence level. A broader age class will make it even more critical to consider interventions that complement vector control, which is supported by our results. The serological criterion as an operational threshold for transmission interruption should be accompanied by vector indicators of decreased presence of domiciliated vectors [17], another potential avenue for further investigation with our model. Besides, PAHO has not yet determined targets for the proportion of *T. cruzi*–infected individuals under care. To this end, our model could help to inform the potential PPC that would need to be achieved and the impact of etiological treatment on transmission and disease burden goals.

There are limitations to this study. The failure to interrupt intradomiciliary vectorial *T. cruzi* transmission in the Americas is likely to be linked not only to a lack of systematic implementation of vector control—particularly in hard-to-reach populations such as rural and indigenous communities [5]—but also to the existence of other *T. cruzi* transmission cycles, such as the presence of sylvatic vector populations in close contact with humans, against which IRS is not as efficacious as with domestic transmission [26]. Vector control has been very efficacious in areas with exclusively domiciliated vectors, as is the case of Central American countries [13]. Regions where vector domiciliation has occurred as a result of close proximity with sylvatic cycles represent a major obstacle for vector control interventions. In our model we represented the role of the sylvatic transmission cycle by adding an external FoI on humans and reservoirs that represents processes taking place outside the domestic cycle. This allows the system always to retain the possibility of *T. cruzi* transmission. Although this can be seen as a simplistic representation of these processes, we have parameterized this using FoI estimates originating from environments where the sylvatic cycle predominates and domiciliated vectors are absent [5, 27]. We acknowledge that this external FoI can be heterogeneous in different environments; further modeling is needed to reflect spatiotemporal changes in this parameter, including diversity of vector species as indicated above [18].

Although we focused on the potential impact on an already well-documented trypanocidal efficacy, we acknowledge that the clinical efficacy of etiological treatment in *T. cruzi–*infected adult populations—essential for reducing Chagas disease burden—has not been proven [9]. In fact, there is a perception that current intervention (antivectorial and antiparasitic) tools against *T. cruzi* are insufficient to eliminate Chagas disease in areas where transmission is currently highest in Central America and northern parts of South America [18].

In conclusion, our model suggests that control programs would benefit from combining vector control with etiological treatment of infected individuals. In terms of vector control, however, its effectiveness will depend on the regional and local vector species involved in or contributing to intradomiciliary transmission and their intrinsic susceptibility to IRS interventions. In terms of etiological treatment, model outputs illustrate that even moderate proportions of annual PPC (10%–20%) would reduce time frameworks for achieving serological thresholds indicative of transmission interruption, infection prevalence in vectors, humans, and reservoirs, and ultimately Chagas disease burden.

## Supplementary Data

Supplementary materials are available at *Clinical Infectious Diseases* online. Consisting of data provided by the authors to benefit the reader, the posted materials are not copyedited and are the sole responsibility of the authors, so questions or comments should be addressed to the corresponding author.

Supplementary Material

## Notes

***Acknowledgments.*** We acknowledge the guidance of the Secretariat of the Neglected Tropical Disease (NTD) Modelling Consortium and the useful discussions held during the annual NTD Modelling Consortium Technical Meetings.

***Disclaimer.*** The views, opinions, assumptions, or any other information set out in this article are solely those of the authors and should not be attributed to the funders or any person connected with the funders. The funders had no role in writing the manuscript or in the decision to submit it for publication.

***Financial support.*** This work was supported by the Bill & Melinda Gates Foundation in partnership with the Task Force for Global Health through the NTD Modelling Consortium (grant number OPP1053230). Z. M. C. received a PhD scholarship from Colciencias, Colombia (grant number 559-11).

***Supplement sponsorship.*** This article appears as part of the supplement “Reaching the 2020 Goals for Nine Neglected Tropical Diseases,” sponsored by the NTD Modelling Consortium.

***Potential conflicts of interest.*** All authors: No reported conflicts of interest. All authors have submitted the ICMJE Form for Disclosure of Potential Conflicts of Interest. Conflicts that the editors consider relevant to the content of the manuscript have been disclosed.
