# Supplementary material for: Complementary Paths to Chagas Disease Elimination: The Impact of Combining Vector Control With Etiological Treatment
Source: Clin Infect Dis. 2018 Jun 1;66(Suppl 4):S293–300. doi: 10.1093/cid/ciy006 (PMC5982731; doi:10.1093/cid/ciy006)
Supplement: Supplementary Material [file ciy006_suppl_supplementary_material.doc]

**Supplementary Materials**

**Complementary Paths to Chagas Disease Elimination: The Impact of Combining Vector Control with Aetiological Treatment**

Zulma M. Cucunubá,^1,2,a^ Pierre Nouvellet,^1,2,a,*^ Jennifer K. Peterson,^3^ Sarah M. Bartsch,^4^ Bruce Y. Lee,^4^ Andrew P. Dobson,^5,a^ Maria-Gloria Basáñez,^1,a^

^1^ London Centre for Neglected Tropical Disease Research, Department of Infectious Disease Epidemiology, School of Public Health, Imperial College London, London, UK

^2^ MRC Centre for Outbreak Analysis and Modelling, Department of Infectious Disease Epidemiology, School of Public Health, Imperial College London, London, UK

^3^ Zoonotic Disease Research Center in Arequipa, Peru and Department of Biostatistics, Epidemiology and Bioinformatics, Perelman School of Medicine, University of Pennsylvania, PA, USA

^4^ Public Health Computational and Operations Research (PHICOR), John Hopkins Bloomberg School of Public Health, Baltimore, MD, USA

^5^ Department of Ecology and Evolutionary Biology, Princeton University, Princeton, NJ, USA

^a^ Contributed equally to this manuscript.

^*^ Present Address: School of Life Sciences, University of Sussex, UK

| **Correspondence to:** | **Alternative contact:** |
| --- | --- |
| Prof María-Gloria Basáñez, PhD | Dr Zulma M. Cucunubá, MD |
| Department of Infectious Disease Epidemiology | Department of Infectious Disease Epidemiology |
| Imperial College London | Imperial College London |
| St Mary’s campus | St Mary’s campus |
| Norfolk Place | Norfolk Place |
| London, W2 1PG, UK | London, W2 1PG, UK |
| Office: +44 (0)20 7594 3295 | Office: +44 (0)20 7594 3229 |
| [m.basanez@imperial.ac.uk](mailto:m.basanez@imperial.ac.uk) | [zulma.cucunuba@imperial.ac.uk](mailto:zulma.cucunuba@imperial.ac.uk) |

**Supplementary Methods**

**Modelling the dynamics of Chagas disease without intervention**

***Triatomine dynamics***

Equations (1) and (2) describe the dynamics of the domiciliated triatomine vector population. The total vector population is represented by$N_{V}$, which comprises uninfected, susceptible vectors,$S_{V}$, and infected (infective) vectors, $I_{V}$. The vector population is regulated by logistic dynamics with per capita growth rate $\left(b_{V}-\mu_{V}\right)$ and carrying capacity $K_{V}$[1] with $b_{V}$the per capita birth rate and $\mu_{V}$the per capita background death rate of vectors,

$\frac{dS_{V}}{dt}=b_{V}N_{V}\left(1-\frac{N_{V}}{K_{V}}\right)-\mu_{V}S_{V}-\beta\left[\frac{c_{a}I_{H}^{a}+c_{i}I_{H}^{i}+c_{c}I_{H}^{c}+c_{r}I_{R}}{N_{H}+N_{R}}\right]S_{V}$ (1)

(2)
$\frac{dI_{V}}{dt}=\beta\left[\frac{c_{a}I_{H}^{a}+c_{i}I_{H}^{i}+c_{c}I_{H}^{c}+c_{r}I_{R}}{N_{H}+N_{R}}\right]S_{V}-\mu_{V}I_{V}$

The carrying capacity of the vectors, $K_{V}$, is proportional to the size of the human population $N_{H}$ on which they feed [1]. Triatomines feed at rate $\beta$ on either humans or animal reservoirs$N_{R}$. The probability of a triatomine becoming infected/ infectious following a bite depends on the type of host it feeds upon, with $c_{a}$, $c_{i}$and $c_{c}$the transmission probabilities when feeding on infected humans who are in the acute, indeterminate or chronic (clinically apparent) phase of the disease, respectively, and $c_{r}$ the probability of transmission when feeding on an infected reservoir.

***Human host dynamics***

The human population is modelled as constant in size, age-structured (yearly age-classes up to 100 years), and in the different stages in disease progression. Following a bite from an infectious domiciliated vector, susceptible humans become infected with probability . While domiciliated transmission is likely to be the most important mode of transmission in highly endemic settings, infection may occur outside of this domiciliated cycle of transmission, e.g. infection by sylvatic vectors. Such additional modes of infection are not modelled explicitly but through a constant ‘external’ force of infection upon humans, denoted$\lambda_{H}$. For simplicity, since we are interested in prevalence patterns rather than in Chagas disease-associated morbidity and mortality, we assume no excess mortality due to infection. Following initial infection, susceptibles enter the acute phase,$I_{H,a}^{a}$, where they remain for an average period equal to $1/\delta$, progressing to the indeterminate stage, $I_{H,a}^{i}$, with no apparent symptoms. From this stage, humans progress at rate $\sigma$ to the determinate, chronic, phase of the disease,, characterised by clinically apparent symptoms. For each age-class we therefore have,

$\frac{dS_{H,a}}{dt}=b_{H}N_{H}-\left[\beta\frac{c_{h}I_{V}}{(N_{H}+N_{R})}+\lambda_{H}\right]S_{H,a}-\mu_{H}S_{H,a}$ if *a* = 0 (3)

$\frac{dS_{H,a}}{dt}=-\left[\beta\frac{c_{h}I_{V}}{(N_{H}+N_{R})}+\lambda_{H}\right]S_{H,a}-\mu_{H}S_{H,a}$ if *a* > 0 (4)

$\frac{dI_{H,a}^{a}}{dt}=\left[\beta\frac{c_{h}I_{V}}{(N_{H}+N_{R})}+\lambda_{H}\right]S_{H,a}-\left(\delta+\mu_{H}\right) I_{H,a}^{a}$ (5)

$\frac{dI_{H,a}^{i}}{dt}=\delta I_{H,a}^{a}-(\sigma+\mu_{H})I_{H,a}^{i}$ (6)

$\frac{dI_{H,a}^{c}}{dt}=\sigma I_{H,a}^{i}-\mu_{H}I_{H,a}^{c}$ (7)

With $N_{H}=\sum_{a} \left(S_{H,a}+I_{H,a}^{a}+I_{H,a}^{i}+I_{H,a}^{c}\right)$ the total human population, $S_{H,a}$ the susceptible human population at age *a*; $I_{H,a}^{i}$ the infected human population at age *a* who are in the acute stage; $I_{H,a}^{i}$the infected human population aged *a* who are in the indeterminate phase, and $I_{H,a}^{c}$ the infected human population aged *a* who are in the chronic stage.

***Reservoir dynamics***

Animal reservoirs are known to play an important role in the transmission dynamics of Chagas disease [2]. While many mammal species can act as competent reservoirs for *Trypanosoma cruzi*, for simplicity, we modelled the reservoirs as a single non-age structured entity,$N_{R}$. The reservoir population was assumed to be constant, of size proportional to the human population, and composed of susceptible reservoirs $S_{R}$ and infected/infectious reservoirs $I_{R}$. Following a bite from an infectious domiciliated vector, reservoirs become infected with probability . Again, the non-domicialiated cycle is not modelled explicitly but through an additional constant ‘external’ force of infection upon the reservoir population denoted $\lambda_{R}$, and there is no excess mortality associated with *T. cruzi* infection in the reservoirs. The following equations therefore describe the dynamics of infection in the reservoir population,

$\frac{dS_{R}}{dt}=b_{R}N_{R}-\mu_{R}S_{R}-\left[\beta\frac{c_{R}I_{V}}{N_{H}+N_{R}}+\lambda_{R}\right]S_{R}$ (9)

$\frac{dI_{R}}{dt}=\left[\beta\frac{c_{R}I_{V}}{N_{H}+N_{R}}+\lambda_{R}\right]-\mu_{R}I_{R}$ (10)

Note that the probability of transmission upon bite from reservoirs to vectors is denoted as $c_{r}$**,** while the probability of transmission from vectors to reservoirs is denoted as $c_{R}$since they have different values. Table A lists the model parameters and provides their values, units and sources.

**Modelling vector control**

We modelled vector control, i.e. indoor residual spraying (IRS) of insecticides, as a sustainable reduction of the density of vectors by a proportion$P_{VC}$. Over the period of implementation of the control strategy, with duration$\Delta_{VC}$, the population of vectors$N_{V}$, as well as their carrying capacity$K_{V}$, are gradually decreased. The decrease is parameterised so as to achieve gradually a final reduction, by$P_{VC}$, of the vector population by the end of the implementation period.

**Modelling treatment of the human population**

We modelled treatment of the human population as a sustainable intervention which reduces the proportion of infected humans through parasite clearance following treatment. The intervention modelled applies to all classes of infectious, i.e. humans in acute, indeterminate and determinate, chronic phases (therefore regardless of their asymptomatic/symptomatic status). Over the period of aetiological treatment implementation, $\Delta_{HT}$, a yearly proportion of all infected humans receives treatment and clears their trypanosomes, therefore returning to the susceptible compartment. This proportion is the equivalent of PPC in the main text (population proportion that achieves parasitological clearance). During the implementation period, the proportion of infected humans treated is gradually (exponentially) increased until the desired level, $1-\exp(-P_{HT})$, is reached.

Table B lists, defines and provides the values used for the interventions modelled.

**Supplementary Tables**

**Supplementary Table A.** Chagas disease model parameters, definition, units and sources

| **Parameter** | **Value (units)** | **Description** | **Source** |
| --- | --- | --- | --- |
| *Parameters pertaining to domiciliated vector dynamics* | | | |
| $b_{V}$ | 10 year^-1^ | Birth rate of triatomine vectors | [3] |
| $\mu_{V}$ | 1.73 year^-1^ | Mortality rate of triatomine vectors | [3] |
| $K_{V}$ | $N_{H} \times C_{V}$ | Carrying capacity of vectors as the product of human population size $N_{H}$ and $C_{V}$ ($C_{V}$from 0.25 to 4) | [4] |
| $\beta$ | 36.5 year^-1^ | Biting rate of domicilated vectors | [1] |
| $c_{a}$ | 0.61 | Probability of transmission upon bite from humans in acute phase to vectors | [5] |
| $c_{i}$ | 0.5 | Probability of transmission upon bite from humans in indeterminate phase to vectors | [6] |
| $c_{c}$ | 0.5 | Probability of transmission upon bite from humans in chronic phase to vectors | [6] |
| $c_{r}$ | 0.5 | Probability of transmission upon bite from reservoirs to vectors | This paper |
| *Parameters pertaining to human host dynamics* | | | |
| $N_{H}$ | 10,000 | Human population size | This paper |
| $b_{H}$ | 0.016 year^-1^ | Human birth rate [equal to death rate to keep population size constant] | This paper |
| $\mu_{H}$ | 0.016 year^-1^ | Human death rate | [7] |
| $c_{h}$ | 0.00058 | Probability of transmission upon bite from vectors to humans | [8] |
| $\delta$ | 8.69 year^-1^ | Progression rate from the acute phase to the indeterminate phase | [9] |
| $\sigma$ | 0.5 year^-1^ | Progression rate from the indeterminate to the determinate, chronic phase | [10] |
| $\lambda_{H}$ | 0.0001 year^-1^ | Constant (external) force of infection from outside the domiciliated cycle acting upon humans | [11] |
| *Parameters pertaining to reservoir host dynamics* | | | |
| $N_{R}$ | $N_{H}\times0.393$ | Reservoir population size | [12] |
| $b_{R}$ | 0.4 year^-1^ | Reservoir birth rate [to keep population size constant] | This paper |
| $\mu_{R}$ | 0.4 year^-1^ | Reservoir death rate | This paper |
| $c_{R}$ | 0.03 | Probability of transmission upon bite from vectors to reservoirs | [13] |
| $\lambda_{R}$ | 0.0002 year^-1^ | Constant (external) force of infection from outside the domiciliated cycle acting upon reservoirs | This paper |

**Supplementary Table B.** Chagas disease intervention model parameters, definition, units and sources

| **Parameter** | **Value (units)** | **Description** | **Source** |
| --- | --- | --- | --- |
| *Parameters pertaining to vector control* | | |  |
| $\Delta_{VC}$ | 0.33 years | Duration of implementation of indoor residual spraying (4 months) | [14] |
| $P_{VC}$ | 0–1 | Decrease in vector density, as a proportion of the vector population removed (0–100%) | This paper |
| *Parameters pertaining to aetiological treatment of humans* | | |  |
| $\Delta_{HT}$ | 1 year | Duration of implementation of aetiological treatment of infected humans in a community. This accounts for the time spent in screening, treatment and monitoring (60 days) | [15,16] |
| $P_{HT}$=  *= PPC* | 0–0.40 | Proportion of infected humans treated. Calculated as a product of:   1. Access to screening (p_T_ = 0–70%) 2. Sensitivity of the tests (p_P_ = 90%) 3. Completion of treatment (p_D_ = 67%) 4. Trypanocidal efficacy (p_E_ = 90%) | This paper  [16,21]  [19]  [16,20]  [17,18] |

**Supplementary Table C.** Sensitivity analysis for the number of years required to achieve the threshold of *T. cruzi* seroprevalence <2% in children aged <5 years for different combinations of intervention scenarios and endemicity levels.

| **Endemicity level** | $C_{V}$ | **Intervention scenario** | **Vector**  **Control (%)** | **PPC (%)** | **No. of years to reach threshold** |
| --- | --- | --- | --- | --- | --- |
| *Very Low* | 0.25 | A | 10 | 1 | 0.1 |
| *Very Low* | 0.25 | B | 50 | 1 | 0.1 |
| *Very Low* | 0.25 | C | 90 | 1 | 0.1 |
| *Very Low* | 0.25 | D | 10 | 10 | 0.1 |
| *Very Low* | 0.25 | E | 50 | 10 | 0.1 |
| *Very Low* | 0.25 | F | 90 | 10 | 0.1 |
| *Low* | 0.5 | A | 10 | 1 | >100 |
| *Low* | 0.5 | B | 50 | 1 | 2.4 |
| *Low* | 0.5 | C | 90 | 1 | 1.4 |
| *Low* | 0.5 | D | 10 | 10 | 2.8 |
| *Low* | 0.5 | E | 50 | 10 | 1.5 |
| *Low* | 0.5 | F | 90 | 10 | 1.1 |
| *Moderate* | 1 | A | 10 | 1 | >100 |
| *Moderate* | 1 | B | 50 | 1 | >100 |
| *Moderate* | 1 | C | 90 | 1 | 5.6 |
| *Moderate* | 1 | D | 10 | 10 | >100 |
| *Moderate* | 1 | E | 50 | 10 | 8.5 |
| *Moderate* | 1 | F | 90 | 10 | 3.9 |
| *High* | 2 | A | 10 | 1 | >100 |
| *High* | 2 | B | 50 | 1 | >100 |
| *High* | 2 | C | 90 | 1 | 10.7 |
| *High* | 2 | D | 10 | 10 | >100 |
| *High* | 2 | E | 50 | 10 | >100 |
| *High* | 2 | F | 90 | 10 | 6.8 |
| *Very high* | 4 | A | 10 | 1 | >100 |
| *Very high* | 4 | B | 50 | 1 | >100 |
| *Very high* | 4 | C | 90 | 1 | 49.1 |
| *Very high* | 4 | D | 10 | 10 | >100 |
| *Very high* | 4 | E | 50 | 10 | >100 |
| *Very high* | 4 | F | 90 | 10 | 11.2 |

Intervention scenarios: A = 10% reduction in vector density and 1% PPC (proportion of parasite clearance); B = 50% reduction in vector density and 1% PPC; C = 90% reduction in vector density and 1% PPC; D = 10% reduction in vector density and 10% PPC; E = 50% reduction in vector density and 10% PPC; F = 90% reduction in vector density and 10% PPC. $C_{V}$ = multiplicative parameter for vector carrying capacity ($N_{H}\times C_{V}$); vector density increases with increasing values of $C_{V}$.

**Sensitivity analysis of the total prevalence**


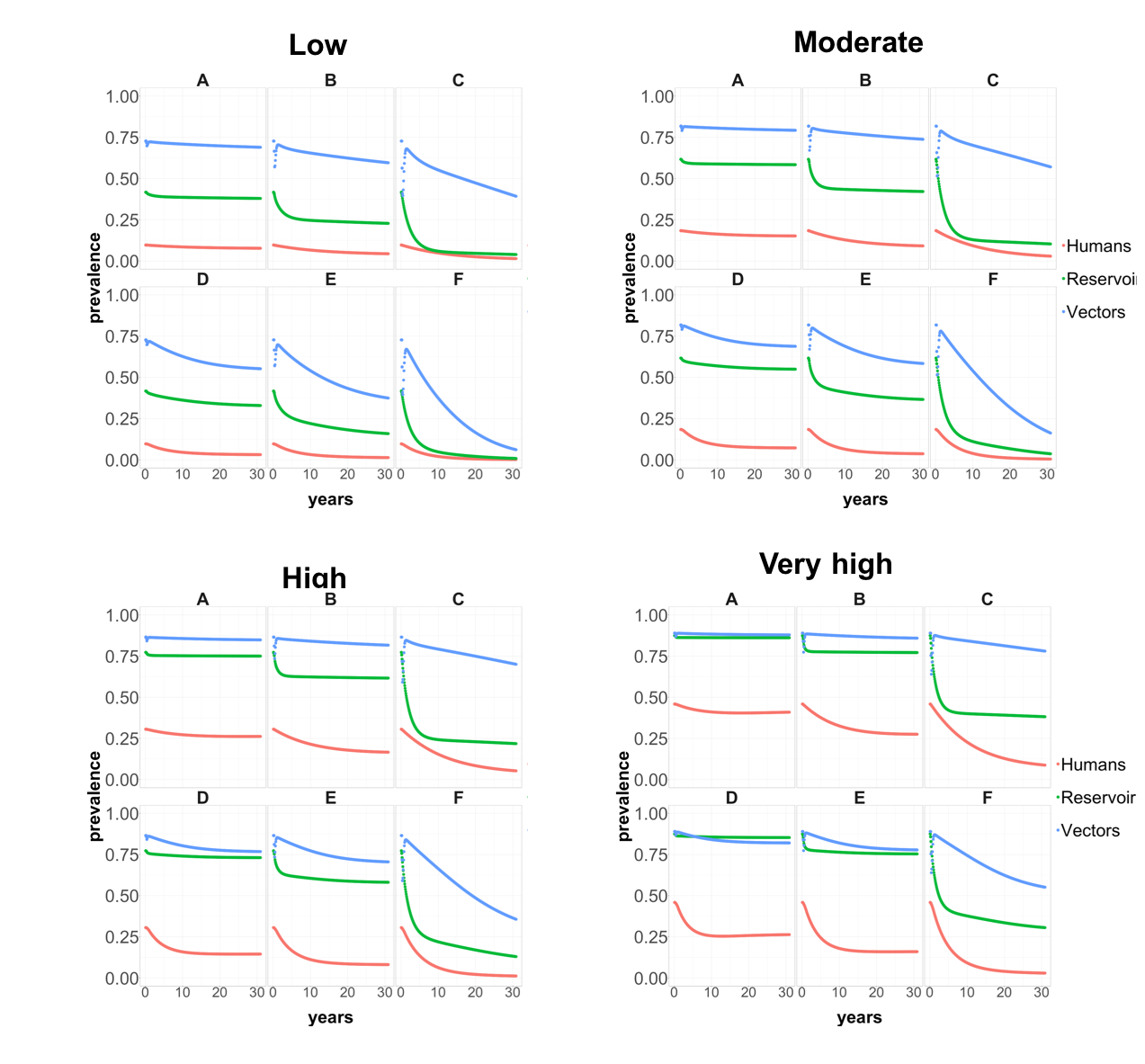


**Supplementary Figure A.** Temporal trends of infection prevalence in humans (red lines), nonhuman mammal hosts (green lines), and domiciliated *Trypanosoma cruzi* vectors (blue lines) over 30 years following the implementation of sustained and continuous control strategies beginning at year 1. In each endemicity scenario (low, moderate, high, and very high), A, B, and C present, respectively, prevalence trends following vector control that leads to 10%, 50%, and 90% reductions in vector density (and 1% PPC). D–F depict prevalence trends following implementation of vector control (same reductions in vector density as above) in combination with an annual 10% proportion of parasitological cure in the population through treatment of the *T. cruzi*–infected human population. The endemicity scenarios were modelled by increasing the carrying capacity of vectors, with the low endemicity scenario corresponding to a vector carrying capacity that is half the size of the human population, and the very high endemicity scenario corresponding to a setting where the vector carrying capacity if four times as high as the total human population size.

**Sensitivity analysis of the age profiles of infection profiles**

**
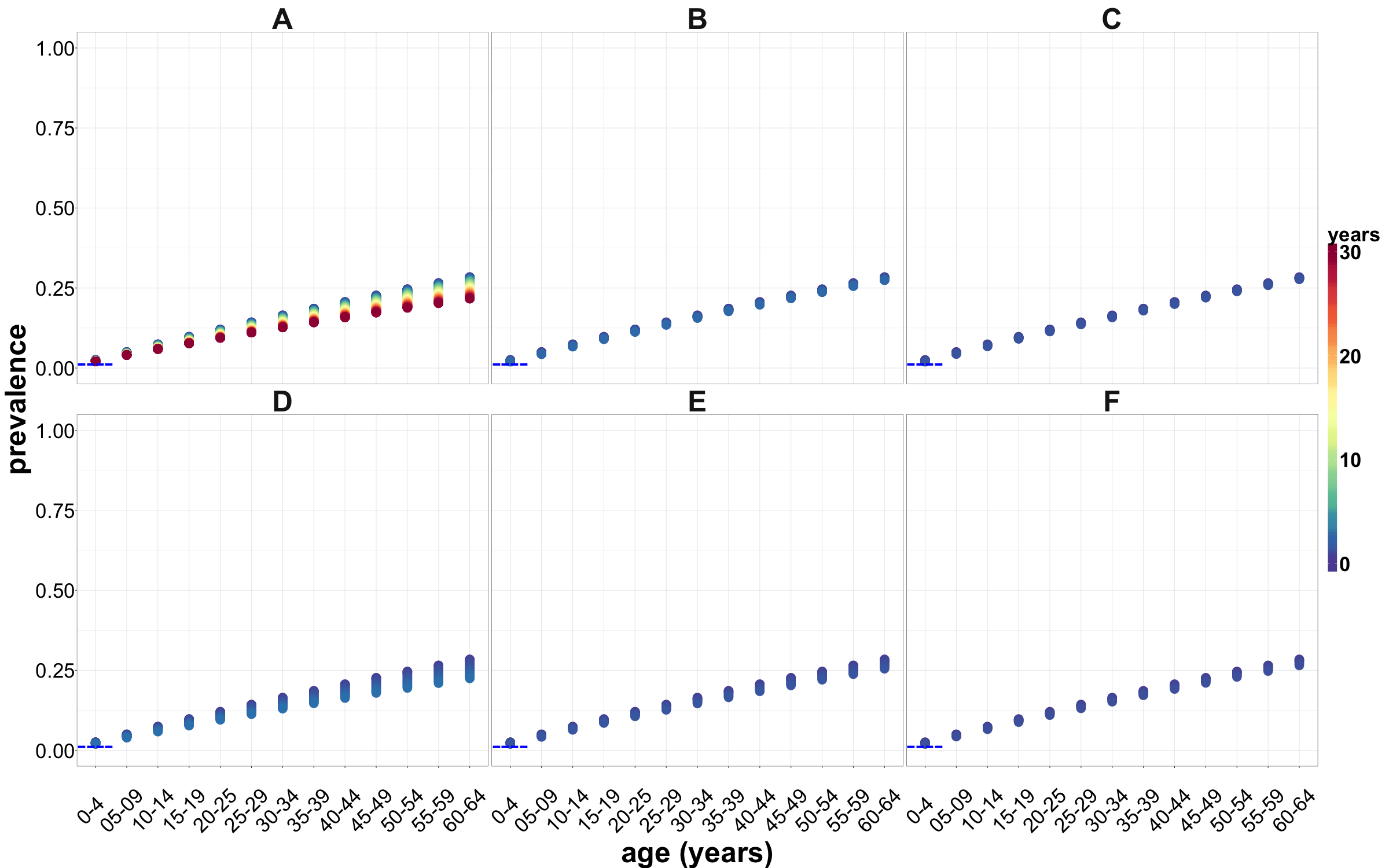
**

**Supplementary Figure B.** Age-specific prevalence of *Trypanosoma cruzi* infection in the human population in a setting of low endemicity ($C_{V}$= 0.50) following the implementation of sustained control strategies. A, B, and C present, respectively, age prevalence profiles corresponding to vector control with annual reductions in vector density of 10%, 50%, and 90% (and 1% PPC). D–F depict human *T. cruzi* infection prevalence following the same reductions in vector density as above in combination with etiological treatment that effects a 10% PPC among the infected population annually. Blue dashed lines indicate the 2% seroprevalence threshold in children under 5. The colour scale represents the number of years of intervention that are necessary to reach the threshold.


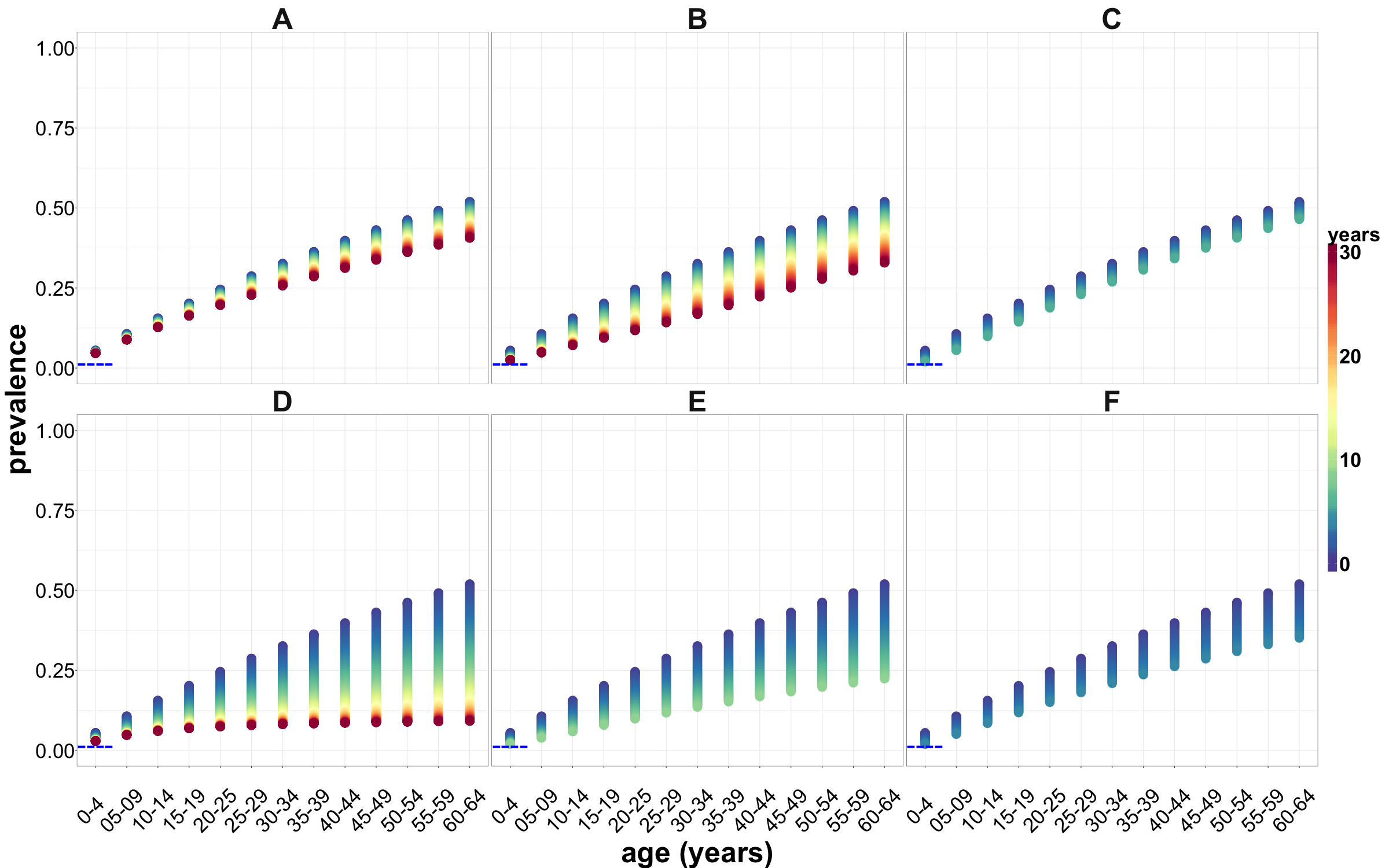


**Supplementary Figure C.** Age-specific prevalence of *Trypanosoma cruzi* infection in the human population in a setting of moderate endemicity ($C_{V}$= 1) following the implementation of sustained control strategies. A, B, and C present, respectively, age prevalence profiles corresponding to vector control with annual reductions in vector density of 10%, 50%, and 90% (and 1% PPC). D–F depict human *T. cruzi* infection prevalence following the same reductions in vector density as above in combination with etiological treatment that effects a 10% PPC among the infected population annually. Blue dashed lines indicate the 2% seroprevalence threshold in children under 5. The colour scale represents the number of years of intervention that are necessary to reach the threshold.


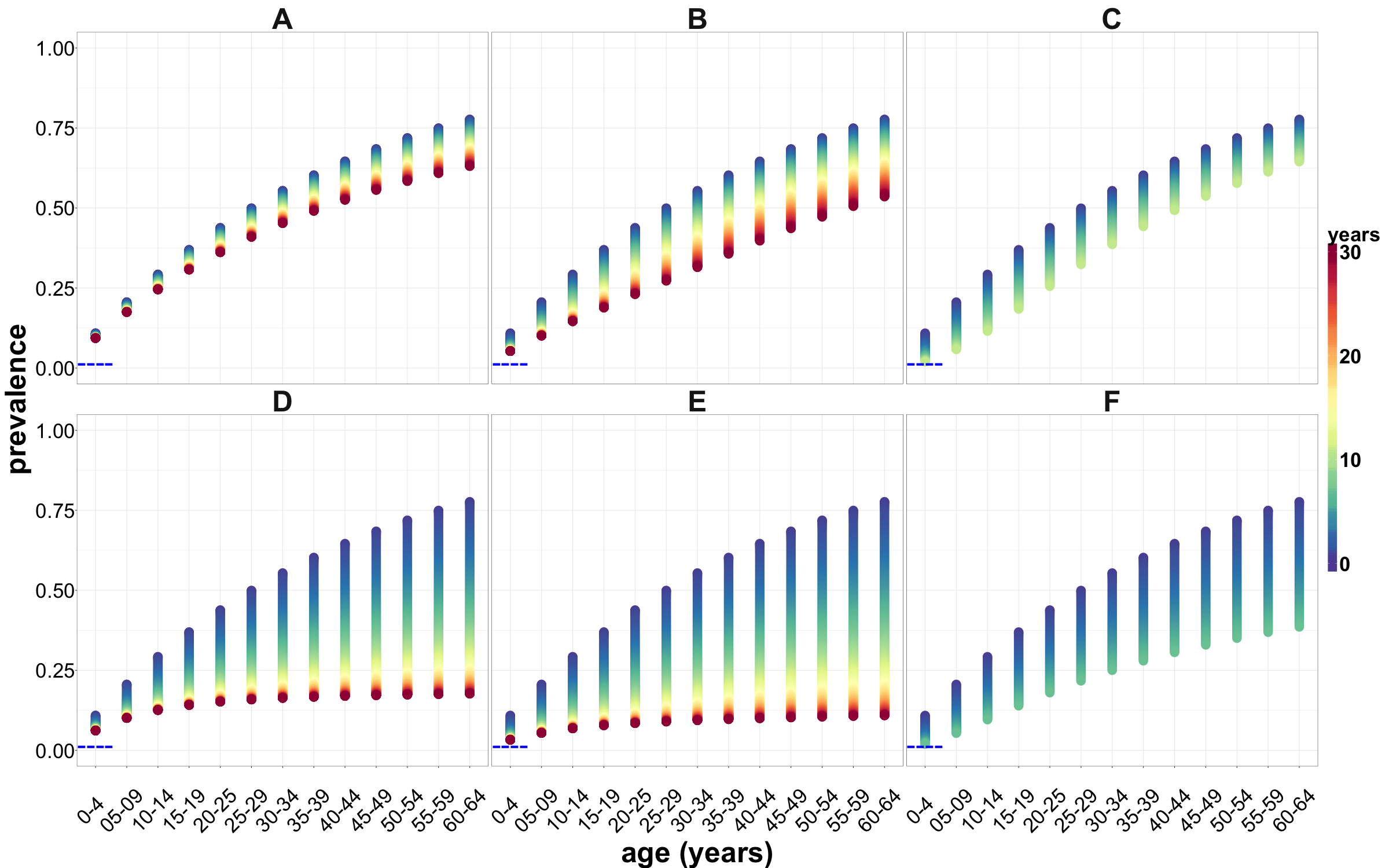


**Supplementary Figure D.** Age-specific prevalence of *Trypanosoma cruzi* infection in the human population in a setting of high endemicity ($C_{V}$= 2) following the implementation of sustained control strategies. A, B, and C present, respectively, age prevalence profiles corresponding to vector control with annual reductions in vector density of 10%, 50%, and 90% (and 1% PPC). D–F depict human *T. cruzi* infection prevalence following the same reductions in vector density as above in combination with etiological treatment that effects a 10% PPC among the infected population annually. Blue dashed lines indicate the 2% seroprevalence threshold in children under 5. The colour scale represents the number of years of intervention that are necessary to reach the threshold.

**
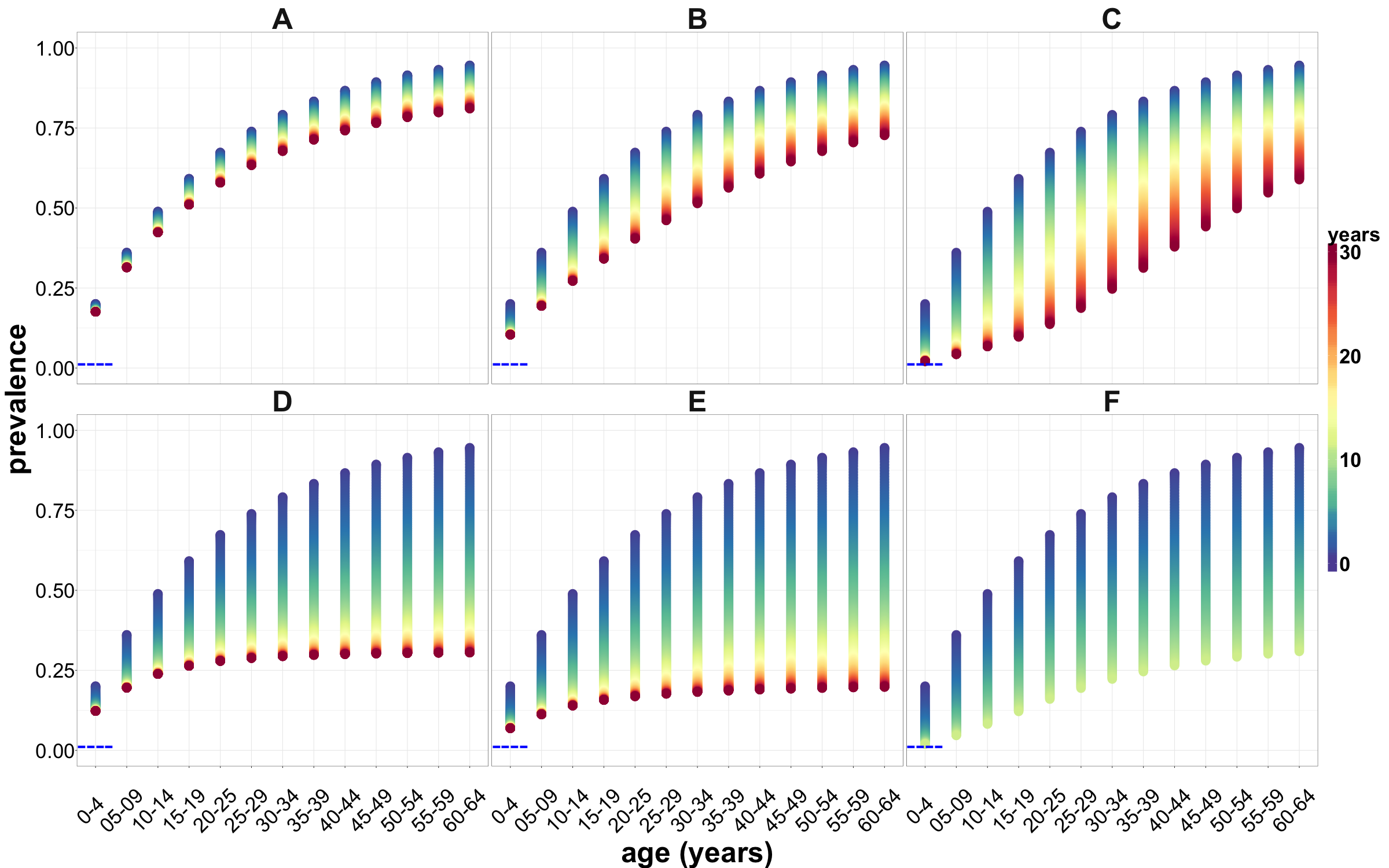
**

**Supplementary Figure E.** Age-specific prevalence of *Trypanosoma cruzi* infection in the human population in a setting of very high endemicity ($C_{V}$= 4) following the implementation of sustained control strategies. A, B, and C present, respectively, age prevalence profiles corresponding to vector control with annual reductions in vector density of 10%, 50%, and 90% (and 1% PPC). D–F depict human *T. cruzi* infection prevalence following the same reductions in vector density as above in combination with etiological treatment that effects a 10% PPC among the infected population annually. Blue dashed lines indicate the 2% seroprevalence threshold in children under 5. The colour scale represents the number of years of intervention that are necessary to reach the threshold.

**Supplementary References**

1. Peterson JK, Bartsch SM, Lee BY, Dobson AP. Broad patterns in domestic vector-borne *Trypanosoma cruzi* transmission dynamics: synanthropic animals and vector control. Parasit. Vectors **2015**; 8:537.

2. Gürtler RE, Cecere MC, Lauricella MA, Cardinal M V, Kitron U, Cohen JE. Domestic dogs and cats as sources of *Trypanosoma cruzi* infection in rural northwestern Argentina. Parasitology **2007**; 134:69–82.

3. Arévalo A, Carranza JC, Guhl F, Clavijo JA, Vallejo GA. Comparación del ciclo de vida de *Rhodnius colombiensis* Moreno, Jurberg & Galvão, 1999 y *Rhodnius prolixus* Stal, 1872 (Hemiptera, Reduviidae, Triatominae) en condiciones de laboratorio. Biomédica **2007**; 27:119–29.

4. Peterson JK, Graham AL, Dobson AP, Chávez OT. *Rhodnius prolixus* life history outcomes differ when infected with different *Trypanosoma cruzi* I Strains. Am. J. Trop. Med. Hyg. **2015**; 93(3):564-72.

5. das Neves Pinto AY, Aldo Valente S, da Costa Valente V, Gomes Ferreira A, Coura JR. Fase aguda da doença de Chagas na Amazônia brasileira. Estudo de 233 casos do Pará, Amapá e Maranhão observados entre 1988 e 2005. Rev. Soc. Bras. Med. Trop. **2008**; 41:602–614.

6. Gurtler RE, Cecere MC, Castañera MB, et al. Probability of infection with *Trypanosoma cruzi* of the vector *Triatoma infestans* fed on human and dogs in northwest Argentina. Am. J. Trop. Med. Hyg. **1996**; 55:24–31.

7. The World Bank. Population, total | Data |. 2016. Available at: http://data.worldbank.org/indicator/SP.POP.TOTL. Accessed 28 August 2017.

8. Nouvellet P, Dumonteil E, Gourbière S. The improbable transmission of *Trypanosoma cruzi* to human: the missing link in the dynamics and control of Chagas disease. PLoS Negl. Trop. Dis. **2013**; 7:e2505.

9. Bern C. Chagas’ Disease. N. Engl. J. Med. **2015**; 373:456–466.

10. Rassi A, Rassi SG. Predictors of mortality in chronic Chagas disease: a systematic review of observational studies. Circulation **2007**; 115:1101–8.

11. Cucunubá Z, Nouvellet P, Conteh L, et al. Modelling historical changes in the force-of-infection of Chagas disease to inform control and elimination programmes: application in Colombia. BMJ Glob. Health **2017**; in press.

12. Bonfante-Cabarcas R, Rodríguez-Bonfante C, Vielma BO, et al. Seroprevalencia de la infección por *Trypanosoma cruzi* y factores asociados en un área endémica de Venezuela. Cad. Saude Publica **2011**; 27:1917–1929.

13. Rabinovich J, Schweigmann N, Yohai V, Wisnivesky-Colli C. Probability of *Trypanosoma cruzi* transmission by *Triatoma infestans* (Hemiptera: Reduviidae) to the opossum *Didelphis albiventris* (Marsupialia: Didelphidae). Am. J. Trop. Med. Hyg. **2001**; 65:125–30.

14. Yoshioka K, Nakamura J, Pérez B, Tercero D, Pérez L, Tabaru Y. Effectiveness of large-scale Chagas disease vector control program in Nicaragua by residual insecticide spraying against triatoma dimidiata. Am. J. Trop. Med. Hyg. **2015**; 93:1231–1239.

15. Bianchi F, Cucunubá Z, Guhl F, et al. Follow-up of an asymptomatic Chagas disease population of children after treatment with nifurtimox (Lampit) in a sylvatic endemic transmission area of Colombia. PLoS Negl. Trop. Dis. **2015**; 10:e0003465.

16. Sartor P, Colaianni I, Cardinal MV, et al. Improving access to Chagas disease diagnosis and etiologic treatment in remote rural communities of the Argentine Chaco through strengthened primary health care and broad social participation. PLoS Negl. Trop. Dis. **2017**; 11:e0005336.

17. Molina I, Gómez i Prat J, Salvador F, et al. Randomized trial of posaconazole and benznidazole for chronic Chagas’ disease. N. Engl. J. Med. **2014**; 370:1899–908.

18. Morillo CA, Waskin H, Sosa-Estani S, et al. Benznidazole and posaconazole in eliminating parasites in asymptomatic *T. cruzi* carriers. J. Am. Coll. Cardiol. **2017**; 69:939–947.

19. Afonso AM, Ebell MH, Tarleton RL, Albajar-Vinas P, Jannin J. A systematic review of high quality diagnostic tests for chagas disease. PLoS Negl. Trop. Dis. **2012**; 6:e1881.

20. Morillo CA, Marin-Neto JA, Avezum A, et al. Randomized trial of benznidazole for chronic Chagas’ cardiomyopathy. N. Engl. J. Med. **2015**; 373:1295–306.

21. Cucunubá ZM, Manne-Goehler JM, Díaz D, et al. How universal is coverage and access to diagnosis and treatment for Chagas disease in Colombia? A health systems analysis. Soc. Sci. Med. **2017**; 175:187-98.
